# Supplementary material for: Motives and Barriers Related to Physical Activity within Different Types of Built Environments: Implications for Health Promotion
Source: Int J Environ Res Public Health. 2022 Jul 24;19(15):9000. doi: 10.3390/ijerph19159000 (PMC9330905; doi:10.3390/ijerph19159000)
Supplement: Supplementary file 1 [file ijerph-19-09000-s001.zip › ijerph-1787838-supplementary.pdf]

Supplementary Table S1. Characteristics of included studies.

Author(s), title, year	Country(s)	Study Type and Method	Target group(s)	Purpose and variable categories	Main results and messages
Abildso, C.G., et al., *Built environment and psychosocial factors associated with trail proximity and use.* American Journal of Health Behavior, 2007.	USA	Quantitative, cross-sectional survey and telephone interview.	788 Adults	Examines the correlation between the local area's built environment, psychosocial factors and subjectively and objectively assessed accessibility and use of local paths.	This study highlights that both the local environment (lack of light, pavement, facilities and other exercise factors) and that psychosocial conditions (lack of time and energy) influence the perceived accessibility of and thus the decision to use local paths. Based on this study's findings, it is recommended that the accessibility/proximity of paths be assessed based on a combination of both objective and subjective measurements.
Anthun K. S., et. al. *Addressing Inequity: Evaluation of an Intervention to Improve Accessibility Quality of a Green Space.* Int. J. Environ. Res. Public Health 2019, 16, 5015, 2019.	Norway	Quantitative, cross-sectional, mixed methods research design. Survey and interview.	2072 Adults	Investigated the use of a green space area and whether and how the area was beneficial for health, social inclusion and physical activity for all socioeconomic groups in a suburban area in Norway.	The study showed significantly increased use of the area from 2015-2018 and that users belonged to different socioeconomic groups. The motivation for using the area was the opportunity to experience nature and to interact socially. The study indicates that factors such as location, availability and designated places for social interaction are important motivating factors for use.
Baert, V., et al., *Motivators and barriers for physical activity in the oldest old: A systematic review.* Ageing Research Reviews, 2011	Several countries	Systematic Review.	Systematic review of 623 studies on the oldest old (80+), whereof 44 studies were included.	To illuminate motives and barriers to physical movement of the oldest elderly (80+)	Intra- and interpersonal factors:<br><u>Motives:</u> Physical health, weight management, physical effects, pain reduction, pleasure and social support from spouse.<br><u>Barriers</u>: Illness, overweight, poor balance, muscle weakness, fear of falling, fear of injury or pain, fear of going out in the evening, lack of time, lack of interest and lack of exercise partners, social resistance, being busy taking care of others<br>Other factors:<br><u>Motives</u>: The content of the training programme.<br><u>Barriers</u>: The content of the training programme, high complexity in the training, finances, temporal placement of the training.
Bamana, A., S. Tessier, and A. Vuillemin,	Belgium, Finland, France,	Quantitative, cross-sectional,	4231 adults, aged 18 years or more	Examines the link between living up to	There is a tendency for people who experience being motivated by their social environment and who observe

Association of perceived environment with meeting public health recommendations for physical activity in seven European countries. Journal of Public Health, 2008	Germany, Italy, Netherlands, Spain, and United Kingdom	questionnaire study		recommendations for physical movement and how opportunities in the local area are experienced	opportunities for physical movement in their local area to be more likely to live up to the recommendations for physical movement than those who are not motivated or do not see opportunities in their local environment. People who experience high social support from their workplace or school are more likely to live up to the recommendations for physical movement. Motivation from local authorities, local sports clubs or others in the local environment showed no effect on living up to physical movement recommendations.
Bauman, A.E., et al., *Correlates of physical activity: why are some people physically active and others not?* The Lancet (British edition), 2012	Several Countries	Review of systematic reviews	Review of 16 systematic reviews	Identifies factors and determinants of physical movement	Perceived ability for physical movement is a determinant of the physical activity level of teenagers, whereas perceived competencies and attitudes are not determinants of that group.<br>Social support, including family support, was related to adolescent physical activity levels, while social and cultural factors and parental activity levels were not correlated with adolescent levels of physical activity.<br><br>Health status and self-efficacy were correlated with physical activity in adults. Opportunities in the local area, such as walkability, experienced traffic safety and green spaces, were related to physically active transport and leisure activity.
Calogiuri, G. and S. Chroni, *The impact of the natural environment on the promotion of active living: An integrative systematic review.* BMC Public Health, 2014	Several countries	Integrative systematic review	Review of 90 studies of young, adults and elderly	Review of existing literature on the relationship between the natural environment and physical movement and the underlying motives for using the natural environment.	The motivation for physical movement in nature is influenced both by individual barriers as well as barriers to accessible nature:<br>-Lack of time, poor health<br>-The subjective experience of the local environment is a strong predictor of physical activity; in particular, the experience of accessibility and security are important.<br>-The degree of accessible nature in the local area is associated with physical movement, but the size of effect varies between studies.<br>-Positive experiences with physical movement can increase intentions to undertake movement.

Calogiuri, G. and L.R. Elliott, *Why do people exercise in natural environments? Norwegian adults' motives for nature-, gym-, and sports-based exercise.* International Journal of Environmental Research and Public Health, 2017	Norway	Quantitative, cross-sectional, questionnaire study	2168 adults	Examines the importance of experiences in nature as motivation for physical movement and what motivates people who exercise outside and those who exercise in gyms.	Experiencing nature was a very important motive for being active, especially for the elderly and those who practice instrumental physical movements, second only to the motive of convenience. However, people who exercise outdoors are motivated more by convenience and experiencing nature than people who exercise in centres and sports clubs. These practitioners are motivated more by physical health and being social with others.
Carlson, J.A., et al., *Interactions between psychosocial and built environment factors in explaining older adults' physical activity.* Preventive Medicine, 2012	USA	Quantitative, cross-sectional, questionnaire study	719 older adults aged 65 years or more<br><br>(Senior Neighborhood Quality of Life Study (SNQLS))	Analyses the association between psychosocial and environmental factors related to physical activity in 719 older adults in Baltimore, Maryland and Seattle, USA.<br>The following parameters were measured:<br>Walkability, access to parks and facilities, moderate to vigorous physical activity, neighbourhood aesthetics, facilities, social support, barriers, transportation	The study showed a correspondence between "walkability" and social support and the level of physical movement.<br>Social support, self-efficacy, and barriers showed correspondence with "walking as transport".<br>In summary, the study showed that living in a supportive environment (vs. non-supportive) was related to a higher level of physical activity.<br>The results support the link between the built environment and psychosocial factors in explaining physical movement in older adults.
Carnegie, M.A., et al., *Perceptions of the physical environment, stage of change for physical activity, and walking among Australian adults.* Research Quarterly for Exercise and Sport, 2002	Australia	Quantitative, cross-sectional, questionnaire study	1200 adults, aged 40-60 years	Examines the relationship between "stages of change" for physical movement and the experience of the physical environment	How the aesthetics and practicalities of the physical environment are perceived is associated with "stage of change" and actual walking behaviour. The study found that those who were considering starting to walk were more negative about the aesthetics and practicalities of the physical surroundings than those who continued walking. Furthermore, the study found that those who walked less than two hours a week were more negative about their physical surroundings than those who walked for more than two hours weekly.

Carraça, E.V., et al., *Lack of interest in physical activity — individual and environmental attributes in adults across Europe: The SPOTLIGHT project.* Preventive Medicine, 2018	5 countries from Europe (Belgium, France, Hungary, and United Kingdom)	Quantitative, cross-sectional, questionnaire study (International Physical Activity Questionnaire - IPAQ)	2014 adults, mean age 51 years	Examines the associations between interest and physical movement after controlling for individual and environmental variables	Interest in physical movement is an important factor in self-reported physical movement, even after adjusting for potential confounders such as perceived barriers to physical movement, structures in the physical environment, lifestyle, self-assessed health, BMI and demographics. Thus, it is important that future studies examine reasons for the lack of interest in physical movement and ways in which interest in and the value of physical movement can be promoted for particular groups and at a population level.
Deelen, I., D. Ettema, and C.B.M. Kamphuis, *Sports participation in sport clubs, gyms or public spaces: How users of different sports settings differ in their motivations, goals, and sports frequency.* PLoS ONE, 2018	Netherlands	Quantitative, cross-sectional, online questionnaire study	910 adults, aged 18-80 years	The study examines how users of different settings differ in relation to "Motivation based on Self-Determination Theory", "Objective" and "Sociodemographic and sport-related characteristics". And how the relationship between motivation and objective is potentially different between participants in physical movement in different settings.	The study concludes that the correlation between motives and participation in sport/physical movement varies from setting to setting. Users of different sports settings varied in terms of personal characteristics, motivation and objective. Among club members, extrinsic goals related to image and intrinsic goals related to skill development, and social affiliation had significant positive associations with sports frequency, whereas in users of informal settings such as parks, health-related goals were positively associated with increased participation. This should be taken into account when designing interventions.
Downward, P., F. Lera-López, and S. Rasciute, *The correlates of sports participation in Europe.* European Journal of Sport Science, 2014	Several EU countries	Qualitative, cross-sectional, interview study	26788 adults aged 15 years and older ("Eurobarometer" European Commission, 2010)	Examines what influences sports participation. In addition to examining common socio-demographic, economic and lifestyle factors, articles also focus on the impact of motivating factors as well as the availability of sports	The decision to participate and frequency of participation in sports is influenced by different factors for men and women. Women are affected by the need to increase self-esteem, whereas men are affected by the desire to join a social community. The provision of sports facilities appears to have a greater impact on men's sports participation, whereas the number of children in the family has a greater impact on women's participation in sport.

				infrastructure and support from the government	
Engbers, L.H. and I.J.M. Hendriksen , *Characteristics of a population of commuter cyclists in the Netherlands: Perceived barriers and facilitators in the personal, social and physical environment.* International Journal of Behavioral Nutrition and Physical Activity, 2010	Netherlands	Quantitative, cross-sectional, questionnaire study	799 Dutch workers, mean age 41 years, 50% women.	Examines perceived barriers and facilitators for cyclists/non-cyclists and personal factors associated with cycling to work	Barriers to cycling to work are perceived distance and travel time. Typical barriers for people who do not cycle are that one is sweaty when arriving at work, the weather and travel time.<br><br>Environmental factors were positively related to more frequent and convenient commuting by bicycle but only for people who already cycled.<br><br>Motives for cycling to work include living close to the workplace, health benefits and working out enough. Facilitators often mentioned in connection with cycling to work: better and more cycle paths, company bicycles, more support management, cycling with others, bicycle racks and showering facilities at work.
Eyler, A.A. and J.R. Vest, *Environmental and policy factors related to physical activity in rural white women.* Women & Health, 2002	USA	Qualitative, cross-sectional interview study (focus groups).	33 women, aged 20-50 years	Identifies environmental and political determinants of physical movement for women in rural areas	Social support was important for the women. Having a training partner, support from family and friends, e.g. that family and friends are themselves active, seemed to be motivating.<br>In relation to barriers, the women mentioned guilt about spending time on activities other than family. Barriers in the surroundings were: lack of pavements, poor occupancy, living close to roads with poor traffic safety, lack of street lighting, fear of exercising alone and too much distance to training facilities.
Gay, J.L., R.P. Saunders, and M. Dowda, *The relationship of physical activity and the built environment within the context of self-determination theory.* Annals of Behavioral Medicine, 2011	USA	Quantitative, cross-sectional, questionnaire study	477 adults, aged 18-85 years, whereof 72.1% were whites, 85.9 % were women	The aim of the study is to analyse the moderating effects of:<br>"Convenience"<br>"Accessibility"<br>"Facilities in preferred locations"<br>"Crime/safety/security" and "characteristics in the residential area" on the	The study showed interaction between neighbourhood characteristics and the three psychological basic needs. There was correlation between accessibility/convenience in the neighbourhood and the perceived competence and belonging of participants.<br>The study indicates that the correlation between physical movement and (autonomy, belonging and competence) differs depending on whether one assesses the accessibility and convenience related to physical movement in the neighbourhood as being high or low.

				satisfaction of the psychological basic needs of Self-Determination Theory (SDT) (autonomy, competence and belonging).	
Gharaveis et al., *A systematic framework for understanding environmental design influences on physical activity in the elderly population.* Understanding environmental design 625, 2020.	Many countries	Literature Review	The Elderly in General	To investigate and understand environmental factors' influence on physical activity for the elderly.	Overall space layout and accessibility to outdoor walkable spaces were addressed to generally promote the moderate levels of physical activity in the elderly population. Space layout and accessibility to outdoor walkable spaces promote moderate levels of physical activity. The important things were: architecture and overall built environment, enhancing factors and barriers: safety, security, assistive devices.
Giles-Corti, B. and R.J. Donovan, *The relative influence of individual, social and physical environment determinants of physical activity.* Social Science & Medicine, 2002	Australia	Quantitative, cross-sectional, questionnaire study	1803 adults, aged 18-59 years	To examine the relative influence of the social environment, physical environment and individual factors on physical movement in leisure time based on the Theory of Planned Behavior (TPB)	The use of facilities depends on distance, easy access and a supportive social environment. "Social support" is essential to being sufficiently physically active.<br>The use of "Open Public spaces" was more sensitive to distance than sports activities such as sports halls and golf facilities. Safety and security in "Open Public Spaces" have implications for use.<br>5 factors are crucial for the level of physical movement in adults aged 18-59:<br>1) Perceived Behavioural control (TPB)<br>2) Behavioural skills (skills related to the given behaviour/ activity)<br>3) Behavioural Intention (TPB)<br>4) Cheering/encouraging physical environment<br>5) Supportive social environment
Heesch, K.C., S. Sahlqvist, and J. Garrard, *Gender differences in recreational and transport cycling: a cross-sectional mixed-methods comparison of*	Australia	Qualitative, cross-sectional, mixed-methods study	1861 adults, aged 18 years or more, whereof 29% were women	To examine the motives and barriers of women and men for using cycling as transport and as a leisure activity	Both sexes described factors such as health and pleasure, as well as the social aspect, as motives for cycling. Barriers to cycling were for both sexes poor road safety. Both men and women preferred to be able to cycle on paths dedicated only to bicycles and not cars.

cycling patterns, motivators, and constraints. International Journal of Behavioral Nutrition and Physical Activity, 2012					Men were more likely to cycle in their spare time and as transport than women, and men also cycled over longer distances.
Keegan, R., et al., *Auditing the socio-environmental determinants of motivation towards physical activity or sedentariness in work-aged adults: A qualitative study.* BMC Public Health, 2016	England	Qualitative, cross-sectional, interview study	15 adults, aged 30-60 years, sound enough to perform high intensity physical activity	The purpose was to identify which social and environmental factors motivated physical movement or inactivity in an adult working population between the ages of 30 and 60. In addition, to analyse in what way these factors interact	The factors that were found to be important in relation to influencing motivation for physical movement were: Spouse/Partner, children, siblings, the family, grandchildren, friends, work colleagues, neighbours, strangers, teammates, instructors, health professionals, employers, government, media and social media, cultural norms and physical environment.<br>Overall, 5 general themes were identified:<br>1) Competence and progress<br>2) Informal influences<br>3) Emotional influences<br>4) Pragmatics and logistics<br>5) Relationships<br>It was found that the same conditions could most often both motivate and demotivate physical movement.
Kelly, S., et al., *Barriers and facilitators to the uptake and maintenance of healthy behaviours by people at mid-life: A rapid systematic review.* PLoS ONE, 2016	Several countries, primarily industrial countries.	Rapid systematic review.<br>Of 16426 titles 28 qualitative studies,11 longitudinal cohort studies and 46 systematic reviews were included	Adults, aged 40-64 years	To analyse barriers and facilitators in relation to health behaviour within the following categories:<br>- Physical movement or inactivity<br>- Diet and nutrition<br>- Weight loss<br>- Smoking<br>- Alcohol	The study found 6 themes of importance for physical movement:<br>"Health and wellness", including:<br>Facilitators: Feeling of "well-being". The desire for health/healthy ageing and weight loss.<br>"Sociocultural factors":<br>Barriers: Lack of time, Lack of knowledge, Social considerations. Facilitators: Supportive surroundings, being a good role model.<br>"Physical Environment":<br>Barriers: Insecurity, the weather, lack of facilities<br>"Accessibility":<br>Barriers: Economy, transport options and limited access.<br>"Psychological factors":<br>Barriers: lack of motivation, lack of Self-efficacy,

					entrenched attitudes and mid-life behaviours.<br>Facilitators: Positive outlook on ageing.
Lee, C., et al., *Neighborhood walking among overweight and obese adults: Age variations in barriers and motivators.* Journal of Community Health, 2013	USA	Quantitative, cross-sectional, questionnaire study	161 overweight adults, aged 20-86 years, mean BMI 32.5	The study analyses barriers and motives for walking in obese adults and additionally analyses variations for specific age groups	The most key environmental barriers to walking for the obese were: lacking road safety, bad weather, inadequate lighting, no shade, stray dogs, poor paving on walkways, no nearby recreation areas, no benches.
Laatikainen, T.E., M. Haybatollahi, and M. Kyttä, *Environmental, individual and personal goal influences on older adults' walking in the Helsinki metropolitan area.* International Journal of Environmental Research and Public Health, 2018	Finland	Quantitative, cross-sectional questionnaire study.<br><br>Citizen science was used for "Online participatory mapping" and "modelling of individual activity spaces" in combination with the questionnaire study.	844 elderly Finnish citizens of Helsinki, aged 55 years or more	Examines residential density and<br>density of walkways and pavements,<br>density of stops, density of public spaces with the possibility of movement for walking as transport	The physical and built environment had an independent effect on the outdoor walking of older adults, independently of individual demographic or psychological personality traits (gender, income level, perceived health, and personal goals).<br><br>Density of habitation, density of walkways, pavements, public transport stops, traffic junctions, and spaces for recreational sporting activities were positively associated with older adults walking for transport.<br><br>The distance between stops had the greatest direct effect in relation to walking in older adults.
McCormack, G.R., et al., *Do motivation-related cognitions explain the relationship between perceptions of urban form and neighborhood walking?*	Canada	Quantitative, cross-sectional, telephone interview and questionnaire study	1967 adults aged 41-60 years, primarily women	Examines the degree to which the Theory of Planned Behavior (TPB) mediates the association between walkability in the local area and walking	Perceived behavioural control was positively associated with walking as transport in the local area but not with leisure walking. One explanation for this could be that leisure walking requires fewer resources and less planning than walking as a mode of transport, thus also less experienced behavioural control.<br>The results suggest that a local area with easy access to

Journal of Physical Activity and Health, 2013		Two independent, cross-sectional samples			shops, transit and other destinations increases perceived behavioural control in relation to walking as transport in the local area. Whereas a high level of security in relation to crime appears to increase perceived behavioural control over leisure walking.
Motl, R.W., et al., *Perceptions of physical and social environment variables and self-efficacy as correlates of self-reported physical activity among adolescent girls.* Journal of Pediatric Psychology, 2007	USA	Quantitative, cross-sectional, questionnaire study	1655 adolescent schoolgirls, mean age 17.7 years	The aim is to examine the direct and indirect effects of perceived access to equipment, local security and social support on self-assessed physical movement over one year	The results show that perceived access to equipment and perceived social support were related to self-assessed self-esteem and that self-esteem and social support were related to self-assessed physical movement for older teenage girls.<br>Equipment perceived as available in the home (e.g. bicycles and balls) or in the local environment (e.g. playgrounds and parks) may promote participation in physical movement for teenage girls by increasing the perceived belief in overcoming barriers. Perceived security in the local area had neither direct nor indirect effect on self-reported physical movement. Results were not influenced by BMI or parental education.
Portegijs E. et. al. *Older Adults' Physical Activity and the Relevance of Distances to Neighborhood Destinations and Barriers to Outdoor Mobility Front.* Public Health 8:335	Finland	Quantitative, cross-sectional survey study	185 older adults, aged 79 to 94 years	The aim of the study was to study associations between older adults' physical activity and self-reported neighbourhood destinations and barriers to outdoor mobility categorised by presence and maximum distance from home.	Attractive neighbourhood destinations for outdoor mobility such as nature, parks and services were associated with higher physical activity. Outdoor mobility barriers, such as street quality and difficult terrain, were associated with markedly lower levels of physical activity.
Santinha G. et. al *Aging and the built environment: is mobility constrained for institutionalized older adults?* Journal of Urbanism:	Portugal	Mixed methods, Cross-sectional study. Observations and semi-	29 professionals, 113 users	This study analyses the location and the neighbourhood environment of residential care facilities and how they influence the walking behaviour of their users.	The general findings were that users value mobility, active lifestyles and social connections, leaving the facilities by active modes of transportation if the built environment of the institutions' surrounding area provides appropriate conditions.<br>Appropriate or motivating conditions are seen as convenience and connectivity.

International Research on Placemaking and Urban Sustainability, 13:4, 431-447, 2020.		structured interviews			Barriers were seen as inadequately sized walking areas, insufficient crossings, the presence of natural and topographic barriers, lighting or signalisation and the number of benches.
Saelens, B.E., et al., *Neighborhood environment and psychosocial correlates of adults' physical activity.* Medicine and Science in Sports and Exercise, 2012	USA	Quantitative, cross-sectional, mixed methods study	2199 adults, aged 20-65 years	The aim of the study is to investigate in what way built environment (e.g. residential density) and experienced (e.g. aesthetics) environmental factors around adult residence have implications for physical movement and reported walking behaviour after adjusting for known psychosocial (e.g. self-efficacy and barriers against physical movement) and demographic correlates (age, gender, ethnicity, education, income, job status) for physical movement	In general, after adjusting for demographic and psychosocial factors, the results show that "neighbourhood built environment" is associated with walking as transport and total moderate to physical movement.<br>The study recommends that policies are generated that support varied action options in residential areas. Including more structured parking and pavements directly outside buildings. It is recommended to build vertically rather than horizontally to be able to establish activities and destinations close to residents.
Simons, D., et al., *Factors influencing mode of transport in older adolescents: A qualitative study.* BMC Public Health, 2013	Belgium	Qualitative, cross-sectional interview study (focus groups).	32 teenagers, aged 17-18 years	An analysis of factors that influence older teenagers' choice of mode of transport	For older young people, the interplay between short travel time, short distance, high autonomy (even being able to choose where and when), good social support (mainly friends), low cost, good access to modes of transport and amenities (cycle paths) and good weather were important factors in choosing active transport over other modes of transport to travel short distances to different destinations. Other well-known factors such as safety, ecology and health did not seem to have much influence on their choice of mode of transport.
Saaty, A.H., et al., *Factors related to engaging in physical*	USA	Mixed methods,	362 female university students, aged 18-	Based on the Theory of Planned Behavior, to analyse motives and	The results showed that "Attitude", "Subjective Norm" and "Perceived behavioural control" along with "weight control" and "attempts to lose weight" were significant

activity: a mixed methods study of female university students. Open Journal of Preventive Medicine, 2015		cross-sectional, questionnaire and focus group interview study	45 years (of whom 74% were aged 18-25 years)	barriers in women to meeting the recommendations for physical movement. 41 questions in connection with:<br>- Socioeconomic status<br>- Participation in physical movement<br>- Attitude<br>- Subjective Norm<br>- Intention<br>- Perceived Behavioural control	predictors of the intention to follow physical movement guidelines." Knowledge of physical movement" was not significant.<br>Focus group interviews showed that barriers to meeting the physical movement recommendations were: lack of accompanying exercise partner, Social support, lack of motivation, time, finances, lack of privacy in the training centre.<br>Social norms were both a barrier and a motivating factor.
Thomson, D. and K. McAdoo, *An exploration into the development of motivation to exercise in a group of male UK regular gym users.* International Journal of Sport and Exercise Psychology, 2016	United Kingdom	Mixed methods, cross-sectional, questionnaire and focus interview study	28 males, aged 21 years or more. All participants were physically active	The study examines the "values, attitudes and experiences" of adult motivated men in relation to regular physical movement in leisure time	The motivation for undertaking regular physical movement is associated with social and environmental stimuli for activity in childhood.<br>Participants' maintenance of a regular physically active lifestyle is contingent on physical movement throughout life becoming a vital element in their own understanding of their identity and their psychological health.<br>The results show that although the specific motives for physical movement varied from individual to individual, the study showed unequivocally that the possibilities of being able to choose, support and social as well as environmental opportunities have a bearing on the motivation for physical movement as an adult and the individual's identity as being physically active.
Ulseth, A.-L.B., *New opportunities – complex motivations: Gender differences in motivation for physical activity in the context of sports clubs and fitness centers.* International Journal of Applied Sports Sciences, 2008	Norway	Quantitative, cross-sectional, questionnaire study	1585 adult members of fitness centres and 1205 adult members of sports clubs, aged 18 or more	To examine gender differences in motivation for physical movement in gyms and sports clubs in a Norwegian context.<br>Variables:<br>1) Fitness (health)<br>2) Appearance<br>3) Enjoyment<br>4) Social factors	Those individuals who primarily state their motivation as being, for example, Enjoyment, social factors and performance are attracted to sports clubs and consist primarily of men.<br>Those individuals who primarily state more "instrumental" motivations such as Fitness, mental recreation and appearance tend to prioritise gyms, and the group consists primarily of women.<br>One of the main findings is that there is a significant difference between which motivations are indicated for

				5) Mental relaxation<br>6) Performance<br>7) Expressiveness<br>The analysis is conducted across a fitness context and a sports club context.<br>Differences in motivation in both contexts and between genders become	being physically active in a fitness context and a sports club context respectively. It is not only against the background of gender difference in the proportion of men and women who participate in the different contexts.<br>Men seem to be motivated more than women by enjoyment and achievement, whereas women more than men appear to be motivated by fitness (health) and appearance.
